# Low dose systemic or intralesional meglumine antimoniate treatment for American tegumentary leishmaniasis results in low lethality, low incidence of relapse, and low late mucosal involvement in a referral centre in Rio de Janeiro, Brazil (2001-2013)

**DOI:** 10.1590/0074-02760160478

**Published:** 2017-12

**Authors:** Lucia Regina Brahim, Cláudia Maria Valete-Rosalino, Liliane de Fátima Antônio, Maria Inês Fernandes Pimentel, Marcelo Rosandiski Lyra, Luiz Eduardo de Carvalho Paes, Ananda Dutra da Costa, Iracema Forni Vieira, Cristina Maria Giordano Dias, Maria Cristina de Oliveira Duque, Mauro Celio de Almeida Marzochi, Armando de Oliveira Schubach

**Affiliations:** 1 Fundação Oswaldo Cruz-Fiocruz, Instituto Nacional de Infectologia Evandro Chagas, Laboratório de Pesquisa Clínica e Vigilância em Leishmanioses, Rio de Janeiro, RJ, Brasil; 2 Universidade Federal do Rio de Janeiro, Departamento de Otorrinolaringologia e Oftalmologia, Rio de Janeiro, RJ, Brasil; 3 Fundação Oswaldo Cruz-Fiocruz, Instituto Oswaldo Cruz, Laboratório de Pesquisas em Leishmanioses, Rio de Janeiro, RJ, Brasil; 4 Secretaria de Estado de Saúde do Rio de Janeiro, Vigilância Epidemiológica, Rio de Janeiro, RJ, Brasil; 5 Secretaria Municipal de Saúde de Timóteo, Timóteo, MG, Brasil; 6 Fundação Oswaldo Cruz-Fiocruz, Instituto Oswaldo Cruz, Laboratório Interdisciplinar de Vigilância Entomológica em Diptera e Hemiptera, Rio de Janeiro, RJ, Brasil

**Keywords:** American tegumentary leishmaniasis, meglumine antimoniate, lethality, relapse, mucosal leishmaniasis

## Abstract

**BACKGROUND:**

American tegumentary leishmaniasis (ATL) is a non-lethal parasitic disease that presents with cutaneous (CL) and mucosal (ML) clinical forms. ATL treatment aims at healing the lesions and preventing the development of the late mucosal form. Systemic meglumine antimoniate (MA) therapy with 10-20 mg Sb^5+^/kg/day is the first choice of treatment. However, alternative therapies using 5 mg Sb^5+^/kg/day or intralesional (IL) MA are the usual regimens at the National Institute of Infectious Diseases (NIID), Rio de Janeiro, Brazil.

**OBJECTIVES:**

To evaluate lethality and the incidence of relapse and development of late ML in CL patients treated at NIID from 2001 until 2013.

**METHODS:**

Data were recovered from records of all ATL patients diagnosed during that period.

**FINDINGS:**

Out of 777 patients, 753 were treated with MA (96.9%). Of those, 89.1% received alternative therapy of 9.9% IL and 79.2% systemic 5 mg Sb^5+^/kg/day. Some patients required 1-3 additional courses of treatment, thus making a total of 997 courses; 85.2% of them were subjected to alternative therapies. Lethality was 0.1%, relapse incidence 5.8%, and late ML incidence 0.25%. As a final outcome for the 777 patients, 95.9% were cured, 0.1% died and 4.0% were not able to follow-up.

**MAIN CONCLUSIONS:**

Alternative MA schedules resulted in low lethality without increase of relapse or late ML incidence.

In Brazil, American tegumentary leishmaniasis (ATL) is a parasitic disease of compulsory notification with registered autochthonous cases in all states. ATL is caused by different dermotropic species of the *Leishmania* genus and transmitted by insects of the Phlebotominae family. *Leishmania (Viannia) braziliensis* is the most frequent and most wide-spread etiological agent in the Brazilian territory. In most cases, ATL caused by *L. (V.) braziliensis* can affect the skin [cutaneous leishmaniasis (CL)] whereas in less than 5% of ATL patients, there is involvement of the mucosa of the upper aerodigestive tract [mucosal leishmaniasis (ML)] (Blum et al. 2012, MS/SVS 2017). It is believed that ML is a secondary form of ATL, caused by dissemination through the blood, and that it can occur several years after healing of the primary cutaneous lesion (Conceição-Silva & Alves 2014, MS/SVS 2017).

According to the Brazilian Ministry of Health, meglumine antimoniate is the first-choice drug for ATL treatment in all endemic areas in this country except where *L. (V.) guyanensis* predominates (MS/SVS 2017). Meglumine antimoniate 1.5 g (Glucantime™, Sanofi Aventis Farmacêutica Ltda, São Paulo, Brazil) is marketed in 5 mL vials containing 405 mg of antimony (Sb^5+^) and it is freely available in the public health network. The recommended dosage for CL treatment is 15 mg Sb^5+^/kg/day for 20 days and for ML treatment is 20 mg Sb^5+^/kg/day for 30 days. In both cases, the drug must be administered via intramuscular (IM) or intravenous (IV) route and, in the case of poor initial response, the treatment should be repeated for another 30 days. If this course of treatment also fails, the use of amphotericin B (desoxycholate or liposomal forms) or pentamidine is recommended as a second choice (MS/SVS 2017). In Brazil, the therapeutic response to meglumine antimoniate is variable depending on the region, even at the recommended doses. However, antimony resistance is not a national public health problem, and 50% to 100% of re-treated patients show a favourable outcome (Tuon et al. 2008, MS/ SVS 2017). The three used drugs are considered effective, but are parenterally administered and sometimes cause serious adverse effects resulting in treatment discontinuation and occasionally in death. Patients’ hepatic, renal, pancreatic, and cardiac functions must therefore be monitored during treatment (WHO 2010, Oliveira et al. 2011, Schubach & Conceição-Silva 2014, MS/SVS 2017).

Although the calculation of meglumine antimoniate dose in mg Sb^5+^/kg/day has been recommended since the 1980s, some textbooks on infectious diseases (Zamith 1976) have suggested the use of one daily vial. Until the 1980s, the use of one daily vial was adopted by the Evandro Chagas National Institute of Infectious Diseases (NIID), Oswaldo Cruz Foundation, Rio de Janeiro (Schubach et al. 2005). A retrospective study regarding patients treated with one daily vial between 1967 and 1982 calculated the range of the received dose from 3.9 to 28.7 mg Sb^5+^/kg/day, depending on the weight of the patients (Schubach et al. 2005). Since the late 1980s, meglumine antimoniate 5 mg Sb^5+^/kg/day IM or IV administered for 30 days constituted the standard CL treatment (Oliveira-Neto et al. 1997a, b, Vasconcellos et al. 2010) and ML treatment at NIID (Oliveira-Neto et al. 2000, Schubach & Conceição-Silva 2014). Additionally, we have used intralesional (IL) meglumine antimoniate for CL treatment, particularly for patients with contraindication to systemic therapy or after treatment discontinuation because of adverse effects or in the treatment of relapsed CL lesions (Oliveira-Neto et al. 1997c, Vasconcellos et al. 2012). These alternative schedules have shown a similar effectiveness to that reported for the 20 mg Sb^5+^/kg/day conventional dose (76.5%), but with less adverse effects (Oliveira-Neto et al. 1997c, Tuon et al. 2008, Vasconcellos et al. 2012, da Silva et al. 2016).

Although clinical trials that evaluate antimonial regimens are necessary (Olliaro et al. 2013), they have been rare. In our literature review, we identified one trial in Bolivia that compared IL meglumine antimoniate with other local treatments for CL caused by *L. (V.) braziliensis* (Soto et al. 2013). However, the authors of this study did not include a control group treated with standard antimonial schedule (20 mg Sb^5+^/kg/day); to establish the sample size they utilised the 80% efficacy result of our previous study (Oliveira-Neto et al. 1997c); and as a “placebo group” they assumed a 10% spontaneous cure rate, as described in Guatemala (Herwaldt et al. 1992). Additionally, the only available study was our own previously published small trial that compared the effects of 5 mg Sb5^+^/kg/day systemic meglumine antimoniate treatment, with 20 mg Sb^5+^/ kg/day in CL treatment (Oliveira Neto et al. 1997b).

Recently, we concluded a single-blind, non-inferiority, randomised controlled trial including 72 patients from Rio de Janeiro that compared an alternative 5 mg Sb^5+^/kg/day dose with the standard 20 mg Sb^5+^/kg/day dose. In the modified intention-to-treat analysis, clinical cure was observed in 77.8% of participants treated with the alternative dose and in 94.4% of the patients treated with the standard dose. However, in the standard dose group we observed more serious adverse events a greater number of adverse events, and a greater number of serious adverse events per participant, in addition to more drug discontinuations, compared to the alternative dose group. Therefore, these results suggest that the alternative dose treatment may be an option especially when toxicity is a concern. Interestingly, 85.7% of all the patients who were originally allocated to the alternative dose antimony group and were followed up after an initial poor therapeutic response were cured after one additional treatment with IL meglumine antimoniate or IM 5 mg Sb^5+^/kg/day (Saheki et al. 2017).

In addition to the epithelialisation of the lesions, CL treatment should enable the healing of definitive cutaneous lesions and the prevention of late mucous membranes involvement. Our early studies on alternative therapeutic schedules with meglumine antimoniate performed during the 1980s included long monitoring of patients regarding absence of re-activation of cutaneous lesions and of late mucosal involvement and were published more than a decade later (Oliveira-Neto et al. 1997a, b, c, Schubach et al. 2005).

In Brazil, between 2001 and 2013, 1,522 deaths out of 337,336 ATL patients were reported, a lethality rate of 4.51 / 1,000 cases (MS 2015). Because ATL per se is not a lethal disease, it is possible that several of these deaths are due to unsuccessful treatment. Despite lethality related to ATL and the positive experience with alternative treatments in Rio de Janeiro, the use of such therapeutic schedules has not been implemented in other regions, because of the assumption that incomplete treatment or the use of Sb^5+^ sub-doses may be related to the occurrence of resistance to antimonials or may be a risk factor for ML development (Blum et al. 2012, MS/SVS 2017). Fortunately, in 2010 the WHO recognised that CL is not a life-threatening condition, that serious complications are not frequent, and that progression to the mucosal form is limited to certain situations. Therefore, safer treatments should be preferred, even if the amount of evidence for their indication is still low (WHO 2010). In 2013, the Pan American Health Organization (PAHO) updated and adapted the WHO recommendations for health service organisation in the Americas and highlighted the need to incorporate the scientific evidence available in each country to the national control programs (OPAS 2013). Recently, the Brazilian Ministry of Health adopted the IL treatment as one option for CL treatment (MS/SVS 2017), implementing the technique recommended by NIID (Duque et al. 2016), as the first option for CL treatment (MS/SVS 2017).

In the present study, we assessed lethality related to meglumine antimoniate therapy and delayed events, such as the incidence of relapse and late development of ML in the ATL cases diagnosed by NIID, likely due to inadequate therapeutic regimens.

## MATERIALS AND METHODS

*Study design and data* - Data was collected from ATL patients from Rio de Janeiro, Brazilian Southeast Region and Brazil (Figure), diagnosed between 2001 and 2013, in three databases: (1) Evandro Chagas National Institute of Infectious Diseases (NIID) - Patient database of a retrospective cohort of the 777 ATL cases identified in the Laboratory of Clinical Research and Surveillance in Leishmaniasis/NIID/Fiocruz, Rio de Janeiro; (2) Rio de Janeiro State - Epidemiological Surveillance Database of the State Health Secretariat of Rio de Janeiro (ASINFO/SVEA/SVS/SES-RJ); (3) Brazilian Southeast Region and Brazil - Notifiable Diseases Information System (SINAN), Ministry of Health, Brazil.

**Figure:**
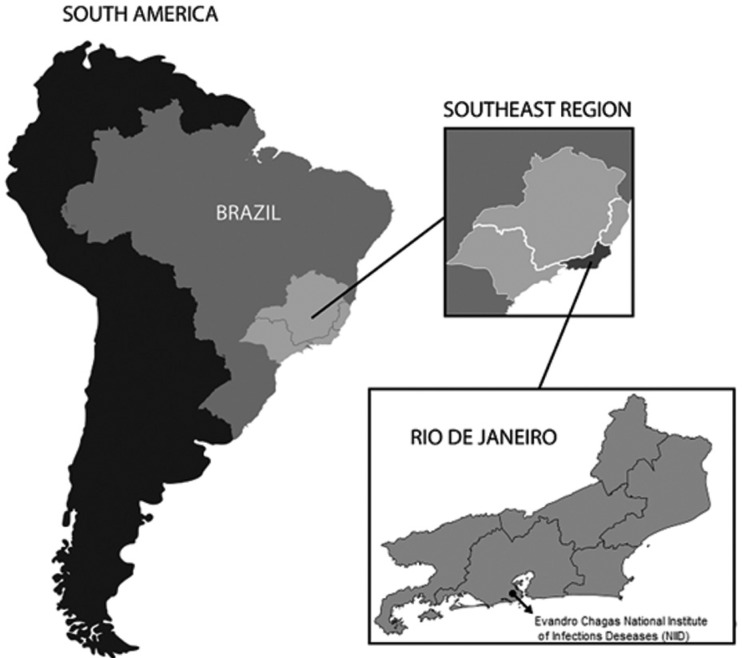
Map of Brazil, highlighting the Southeast Region, the State of Rio de Janeiro, and the location of the Evandro Chagas National Institute of Infectious Diseases (NIID) in the city of Rio de Janeiro.

The indices from the cohorts were evaluated only in the database of NIID patients and were calculated as percentages: “incidence of relapse” (% of naive patients first treated at NIID for the same clinical form and then relapsed after the treatment); “incidence of mucosal leishmaniasis” (% of patients treated for CL at NIID that developed into ML); “lethality” (% death); “prevalence of relapse” (% of patients that relapsed after treatment); and “prevalence of mucosal leishmaniasis” (% of ML cases).

In addition, we assessed the lethality, the relapse prevalence and prevalence of ML in the Rio de Janeiro State, Southeast Region and Brazil to examine whether the alternative schedules with meglumine antimoniate used at NIID could adversely compromise these indices in relation to other states in the Southeast Region, where such alternative schemes are not used. The indices “lethality”, “prevalence of relapses” and “prevalence of mucosal leishmaniasis” regarding the Rio de Janeiro State were retrieved from the ASINFO/SVEA/SVS/SES-RJ database, and those from the Southeast Region and from Brazil were retrieved from the SINAN database and were calculated for every 1,000 cases of ATL. In the estimate, the following data obtained in the fields # 33, 39 and 56 of the notification form of the ASINFO/SVEA/SVS/SES-RJ and SINAN databases were considered: (i) “Lethality”: positive notification for “death by ATL” or “death by other causes” in field # 56 - “Case evolution”; (ii) “Prevalence of relapse”: positive notification for “relapse” in field # 39 - “Entry type”; (iii) “Prevalence of mucosal leishmaniasis”: positive notifications for “yes” in “mucosal lesion” in field # 33 - “Presence of cutaneous and mucosal lesion”.

*Therapy* - At NIID, as a rule, the first choice of therapy was IM or IV meglumine antimoniate 5 mg Sb^5+^/kg/ day (for 30 days for CL and up to 120 days for ML), or IL meglumine antimoniate for CL, as previously described (Schubach & Conceição-Silva 2014, Duque et al. 2016). Meglumine antimoniate schedule for patients included in an arm of a clinical trial during the same study period was IM 20 mg Sb^5+^/kg/day for 20 days (Saheki et al. 2017). Amphotericin B or pentamidine were administered according to the recommendations of the Brazilian Ministry of Health guidelines (MS/SVS 2017).

In other sites in Rio de Janeiro State, Southeast Region and Brazil, we assumed that, as a rule, the first choice of treatment was IM or IV meglumine antimoniate 10 – 20 mg Sb^5+^/kg/day for a duration of 20 – 30 days, as recommended by the Brazilian guidelines (MS/SVS 2017).

*Ethics* - This study was approved by the Committee of Ethics in Research of the Evandro Chagas National Institute of Infectious Diseases (#CAAE 17222113.2.0000.5262).

## RESULTS

Between 2001 and 2013, 777 ATL patients were treated at NIID: 669 (86.1%) were from the State of Rio de Janeiro, 24 (3.1%) from other states of the southeast region, 37 (4.8%) from the Brazilian North Region, 41 (5.3%) from the Northeast Region, 3 (0.4%) from the Center-West Region, 2 (0.2%) cases from French Guiana and 1 (0.1%) from Israel.

These 777 patients comprised of 581 (74.8%) CL and 196 (25.2%) ML cases, corresponding to 76.2% of the ML cases diagnosed in Rio de Janeiro State in the same period.

At NIID, meglumine antimoniate was the first-choice drug and was administered to 753 (96.9%) patients. Of those, 692 (89.1%) were treated with an alternative therapeutic schedule: 615 (79.2%) patients received IM or IV 5 mg Sb^5+^/kg/day, and 77 (9.9%) were treated with IL meglumine antimoniate. The 20 mg Sb^5+^/kg/day standard dose was used in 61 (7.8%) patients. Amphotericin B (desoxycholate or liposomal forms) was the drug of choice for the initial treatment of 17 (2.2%) patients with contraindication to meglumine antimoniate. In cases of poor therapeutic response or that relapsed after an apparent good initial response, one to three additional courses of treatment were necessary thus making a total of 997 treatment courses in the 777 patients (Table I). Considering all 220 retreatments, the time interval between courses of treatment (1-2, 2-3 and 3-4) ranged from one day to 39 months, with a median of 3.6 months.

**TABLE I:** Number of treatments applied in American tegumentary leishmaniasis cases notified by the National Institute of Infectious Diseases, Oswaldo Cruz Foundation, Rio de Janeiro, Brazil, between 2001-2013, according to the used therapeutic scheme

Therapeutic scheme	Treatment number				
	1st n (%)	2nd n (%)	3rd n (%)	4th n (%)	total n (%)
Intralesional meglumine antimoniate	77 (9.9)	37 (24.5)	6 (12.0)	2 (10.5)	122 (12.2)
Meglumine antimoniate 5 mg Sb^5+^/kg/day	615 (79.2)	85 (56.3)	22 (44.0)	6 (31.7)	728 (73.1)
Meglumine antimoniate 20 mg Sb^5+^/kg/day	61 (7.8)	1 (0.7)	1 (2.0)	1 (5.2)	64 (6.4)
Amphotericin B	17 (2.2)	19 (12.5)	19 (38.0)	8 (42.1)	63 (6.3)
Pentamidine	3 (0.4)	7 (4.6)	-	2 (10.5)	12 (1.2)
Other	4 (0.5)	2 (1.4)	2 (4.0)	-	8 (0.8)
Total	777 (100)	151 (19.4)	50 (6.4)	19 (2.4)	997 (128.3)

n (%): absolute number of cases (percentage).

Of the 175 relapsed cases diagnosed in Rio de Janeiro State in the same period, 144 (82.3%) were attended at NIID, corresponding to 18.5% of the 777 patients treated. Out of these 144 relapsed cases, 99 (68.8%) were admitted at the NIID with already relapsed lesions after a previous treatment received in another health service and 45 (31.2%) naive patients relapsed after the first treatment received at NIID.

As a rule, patients with poor therapeutic response or those that relapsed received one to three more courses of IM or IV 5 mg Sb^5+^/kg/day or IL meglumine antimoniate, or amphotericin B. Most patients required re-treatment with meglumine antimoniate 5 mg Sb^5+^/kg/day; however only 6 (31.7%) of the 19 patients required the fourth treatment, while 8 (42.1%) of these patients required the use of amphotericin B. Only three patients were re-treated with the standard dose of 20 mg Sb^5+^/kg/day. Overall, meglumine antimoniate was the drug most frequently used (914 – 91.7% - out of the 997 courses of treatment), followed by amphotericin B in 63 (6.3%), whereas other drugs including pentamidine, fluconazole or itraconazole were used in the remaining 20 cases (2%) (Table I).

One (0.1%) female CL patient out of the 777 ATL individuals treated at NIID, who was 43 years old and had hypertension and type II diabetes, stopped antimonial therapy 20 mg Sb^5+^/kg/day due to asymptomatic hyperlipasemia and died soon after interruption of therapy due to diabetic ketoacidosis associated with sepsis. Forty-five ATL patients treated with IM or IV 5 mg Sb^5+^/kg/ day or IL meglumine antimoniate relapsed (incidence of relapse = 5.8%): 36 CL patients relapsed between one and 60 months (median = seven months) after the end of the treatment, and 9 ML patients between one and 36 months (median = five months). Two other CL patients developed ML (incidence of ML = 0.25%): one was treated with meglumine antimoniate 5 mg Sb^5+^/kg/ day via intramuscular route and developed ML after 13 months, and the other received IL meglumine antimoniate and developed ML after one month.

As a final outcome for the initial 777 patients, 745 (95.9%) were cured, one (0.1%) died and 31 (4%) abandoned follow-up.

The lethality, prevalence of relapse and prevalence of mucosal leishmaniasis from Rio de Janeiro State, Southeast Region and Brazil were calculated for every 1,000 cases of ATL and are shown in (Table II).

**TABLE II:** Lethality, prevalence of relapses and prevalence of mucosal leishmaniasis (ML) for every 1,000 cases of American tegumentary leishmaniasis (ATL) notified between 2001-2013 at Rio de Janeiro State, Southeast Region, and Brazil

	Lethality	Relapse prevalence	ML prevalence	Number of notified ATL cases
Rio de Janeiro State	4.94	61.75	90.68	2,834
Southeast Region	13.46	58.22	112.18	31,412
Brazil	4.51	44.34	62.51	337,336

Sources: Notifiable Diseases Information System (SINAN), Brazilian Ministry of Health; and Advisory Services for Epidemiological and Environmental Surveillance of Rio de Janeiro (ASINFO/SVEA/SVS/SES/RJ).

## DISCUSSION

Since the late 1980s, meglumine antimoniate via IL or 5 mg Sb^5+^/kg/day, via IM or IV, was the standard treatment for ATL patients at NIID, and these alternative schedules have been effective with minimum adverse effects (Oliveira-Neto et al. 1997a, b, c, Oliveira-Neto et al. 2000, Vasconcellos et al. 2010, 2012, Schubach & Conceição-Silva 2014). In the last three decades, the conventional treatment with meglumine antimoniate 20 mg Sb^5+^/kg/day has not been commonly used at NIID for ATL treatment (Schubach & Conceição-Silva 2014), independently of the Brazilian Region or State of origin of these patients (Antônio et al. 2014). During the 13 years of the study period, only 6.4% of the patients were treated with meglumine antimoniate 20 mg Sb^5+^/kg/day, and for of the vast majority of these individuals, the use of this dose was required due to their participation in clinical trials for CL (Saheki et al. 2017) or for ML (ongoing).

Here, we evaluated the following indices related to the therapy with meglumine antimoniate in cases of ATL reported by NIID: lethality, incidence of relapse and development of ML. The first one is usually a consequence of the toxicity of meglumine antimoniate, and the other two are delayed events that may be attributed to inadequate therapeutic regimens. Because NIID is a centre where the most serious and complicated cases from several Brazilian Regions are referred to, we hypothesized that we would observe high lethality; however, high lethality was not observed. In addition, if the alternative schedules of meglumine antimoniate were ineffective, we would expect to find high incidence of relapse and development of late ML.

Although the prevalence of relapsed patients treated at NIID represents the absolute majority of such cases diagnosed in Rio de Janeiro, the incidence of relapse among patients initially treated at NIID, for the same clinical form, did not differ from that reported in other studies. A previous study examining CL patients treated at NIID with 5 mg Sb^5+^/kg/day meglumine antimoniate for 30 days, between January 1989 and December 2009, showed that in 14% of the cases there was a poor therapeutic response to meglumine antimoniate (there was no healing after the initial treatment) (Antônio et al. 2014). In some other series of cases with patients treated with alternative schedules of meglumine antimoniate the incidence of poor therapeutic response varied between 16 and 20%. Most of those patients were monitored from 1 to 14 years and, of those, all the patients that remained were cured (Oliveira-Neto et al. 1997a, b, c, Vasconcellos et al. 2010, 2012). These findings were similar to those reported by others (19% to 58%) (Deps et al. 2000, Teixeira et al. 2008) and support the effectiveness of low-dose (5 mg Sb^5+^/kg/day) and IL treatment with meglumine antimoniate.

In Brazil, therapeutic failure is defined as a poor therapeutic response to two consecutive schedules of meglumine antimoniate 10 to 20 mg Sb^5+^/kg/day as a standard dose for CL. If the second treatment fails, it is recommended to use amphotericin B or pentamidine (MS/SVS 2017). However, at NIID, we generally treat patients with poor initial therapeutic response or who relapse after an apparent good initial therapeutic response with IM or IV 5 mg Sb^5+^/kg/day or IL meglumine antimoniate, once or twice before attempting treatment with another drug. Amphotericin B was the second mostly used drug. However, its use as a therapeutic option increased from 2.2% in the first to 42.1% in the fourth treatment course. There was a rare requirement for the use of a third drug, such as pentamidine (1.2%) (Pimentel et al. 2011).

Of note, during the 13 years of this study, the absolute majority of ML cases diagnosed in Rio de Janeiro were treated at NIID. Despite that, only 0.25% of the patients initially treated for CL developed ML. At NIID, all ATL patients, regardless of mucous membranes complaint, are systematically evaluated by endoscopic methods that allow early diagnosis and treatment of mucosal lesions, including patients erroneously referred to initially as CL cases (Costa et al. 2014). This may explain why the concomitant form (simultaneous presence of cutaneous and mucosal lesions) is the most common form of ML at NIID and consequently, in Rio de Janeiro State. Other authors reported that the late mucosal form is the most frequent (MS/SVS 2017). It is possible that incipient mucosal lesions, not identified and not treated early, could evolve to mucosal lesions diagnosed later.

As a rule, lethality, prevalence of relapse, and prevalence of ML calculated for the State of Rio de Janeiro were similar to those of the Southeast Region where Rio de Janeiro is located. NIID reported about 1/4 of the ATL cases reported in the State of Rio de Janeiro, including the absolute majority of the more complicated cases (such as prevalent relapses and ML cases). Therefore, we hypothesize that the alternative schedules with meglumine antimoniate used at NIID did not adversely compromise these indices of the State of Rio de Janeiro in relation to the other states in the Southeast Region, where such alternative schemes are not used.

In contrast, the three indices were higher in the Southeast Region than in Brazil. These indices probably reflect both the treatment of more severe cases in reference centres with better diagnosis conditions and the better epidemiologic surveillance services of that Region for the notification of cases. In another publication, which data of ATL notification from different Brazilian Regions were included, from 2002 to 2009, it was observed that areas with lower prevalence of infection such as the Southeast Region presented higher prevalence of ML. It was suggested that the lower prevalence of ML in old endemic areas with high infection prevalence may be explained by better adaptation between the host, the parasite, and the vector (Bedoya-Pacheco et al. 2011).

Our findings suggest that the alternative schedules with meglumine antimoniate used in NIID resulted in low lethality, without increase of the incidence and the prevalence of relapse or the development of late ML at NIID and in the whole State of Rio de Janeiro.

## References

[B1] Antônio LF, Fagundes A, Oliveira R, Pinto P, Vasconcellos EFC, Bedoya-Pacheco SJ (2014). Montenegro skin test and age of skin lesion as predictors of
treatment failure in cutaneous leishmaniasis. Rev Inst Med Trop São Paulo.

[B2] Bedoya-Pacheco SJ, Araujo-Melo MH, Valete-Rosalino CM, Pimentel MIF, Conceição-Silva F, Schubach AO (2011). Endemic tegumentary leishmaniasis in Brazil: correlation between
level of endemicity and number of cases of mucosal disease. Am J Trop Med Hyg.

[B3] Blum J, Lockwood DN, Visser L, Harms G, Bailey MS, Caumes E (2012). Local or systemic treatment for New World cutaneous
leishmaniasis? Re-evaluating the evidence for the risk of mucosal
leishmaniasis. Int Health.

[B4] Conceição-Silva F, Alves CA (2014). Leishmanioses do Continente Americano.

[B5] Costa DCS, Palmeiro MR, Moreira JS, Martins ACC, Silva AF, Madeira MF (2014). Oral manifestations in the American tegumentary
leishmaniasis. PloS ONE.

[B6] da Silva RE, Toledo A, Senna MC, Rabello A, Cota G (2016). Intralesional meglumine antimoniate for the treatment of
localised cutaneous leishmaniasis: a retrospective review of a Brazilian
referral centre. Mem Inst Oswaldo Cruz.

[B7] Deps PD, Viana MC, Falqueto A, Dietze R (2000). Avaliacão comparativa da eficácia e toxicidade do antimoniato de
N-metil-glucamina e do estibogluconato de sódio BP88©no tratamento da
leishmaniose cutânea localizada. Rev Soc Bras Med Trop.

[B8] Duque MCO, Vasconcellos EFC, Pimentel MIF, Lyra MR, Bedoya-Pacheco SJ, Marzochi MCA (2016). Standardization of the technique for the treatment of cutaneous
leishmaniasis with meglumine antimoniate via the intralesional
route. Rev Soc Bras Med Trop.

[B9] Herwaldt B, Arana B, Navin T (1992). The natural history of cutaneous leishmaniasis in
Guatemala. J Infect Dis.

[B10] MS - Ministério da Saúde [Internet] (2001-2013). Sistema de Informação de Agravos de Notificação SINAN.

[B11] MS/SVS - Ministério da Saúde/Secretaria de Vigilância em
Saúde (2017). Departamento de Vigilância das Doenças Transmissíveis. Manual de
vigilância da leishmaniose tegumentar [electronic resource].

[B12] Oliveira LF, Schubach AO, Martins MM, Passos SRL, Oliveira RV, Marzochi MCA (2011). Systematic review of the adverse effects of cutaneous
leishmaniasis treatment in the New World. Acta Trop.

[B13] Oliveira MP, Mattos M, Pirmez C, Fernandes O, Goncalves-Costa SC, Souza CF (2000). Mucosal leishmaniasis (“espundia”) responsive to low dose of
N-methyl glucamine (Glucantime) in Rio de Janeiro, Brazil. Rev Inst Med Trop São Paulo.

[B14] Oliveira MP, Schubach A, Mattos M, Goncalves-Costa SC, Pirmez C (1997a). A low dose antimony treatment In 159 patients with American
cutaneous leishmaniasis. Extensive follow-up studies (up to 10
years). Am J Trop Med Hyg.

[B15] Oliveira MP, Schubach A, Mattos M, Goncalves-Costa SC, Pirmez C (1997b). Treatment of American cutaneous leishmaniasis: a comparison
between low dosage (5mg/kg/day) and high dosage (20mg/ kg/day) antimony
regimens. Pathol Biol.

[B16] Oliveira MP, Schubach A, Mattos M, Goncalves-Costa SC, Pirmez C (1997c). Intralesional therapy of American cutaneous leishmaniasis with
pentavalent antimony in Rio de Janeiro, Brazil - an area of
*Leishmania (V) braziliensis*
transmission. Int J Dermatol.

[B17] Olliaro P, Vaillant M, Arana B, Grogl M, Modabber F, Magill A (2013). Methodology of clinical trials aimed at assessing interventions
for cutaneous leishmaniasis. PLoS Negl Trop Dis.

[B18] OPAS - Organización Panamericana de la Salud (2013). Leishmaniases en las Américas: recomendaciones para el
tratamiento.

[B19] Pimentel MI, Baptista C, Rubin EF, Vasconcellos EFC, Lyra MR, Salgueiro MM (2011). American cutaneous leishmaniasis caused by *Leishmania
(Viannia) braziliensis* resistant to meglumine antimoniate, but
with good response to pentamidine: a case report. Rev Soc Bras Med Trop.

[B20] Saheki MN, Lyra MR, Bedoya-Pacheco SJ, Antônio LF, Pimentel MIF, Salgueiro MM (2017). Low versus high dose of antimony for American cutaneous
leishmaniasis: a randomized blind non-inferiority trial in Rio de Janeiro,
Brazil. PLoS ONE.

[B21] Schubach AO, Conceição-Silva F, Conceição-Silva F, Alves CA (2014). Estado da Arte no tratamento da leishmaniose
tegumentar Americana no Brasil. Leishmanioses do Continente Americano.

[B22] Schubach AO, Marzochi KB, Moreira JS, Schubach TM, Araújo ML, Vale AC (2005). Retrospective study of 151 patients with cutaneous leishmaniasis
treated with meglumine antimoniate. Rev Soc Bras Med Trop.

[B23] Soto J, Rojas E, Guzman M, Verduguez A, Nena W, Maldonado M (2013). Intralesional antimony for single lesions of Bolivian cutaneous
leishmaniasis. Clin Infect Dis.

[B24] Teixeira AC, Paes MG, Guerra JO, Prata A, Silva-Vergara ML (2008). Failure of both azithromycin and antimony to treat cutaneous
leishmaniasis in Manaus, AM, Brazil. Rev Inst Med Trop São Paulo.

[B25] Tuon FF, Amato VS, Graf ME, Siqueira AM, Nicodemo AC, Amato V (2008). Treatment of New World cutaneous leishmaniasis - a systematic
review with a meta-analysis. Int J Dermatol.

[B26] Vasconcellos ECF, Pimentel MIF, Schubach AO, Oliveira RVC, Azeredo-Coutinho RB, Conceição-Silva F (2012). Intralesional meglumine antimoniate for treatment of cutaneous
leishmaniasis patients with contraindication to systemic therapy from Rio de
Janeiro (2000 to 2006). Am J Trop Med Hyg.

[B27] Vasconcellos EFC, Schubach AO, Valete-Rosalino CM, Coutinho RS, Conceição-Silva F, Salgueiro MM (2010). American tegumentary leishmaniasis in older adults: 44 cases
treated with an intermittent low-dose antimonial schedule in Rio de Janeiro,
Brazil. J Am Geriatr Soc.

[B28] WHO - World Health Organization (2010). Control of the leishmaniases. WHO Technical Report Series. Report No.
949.

[B29] Zamith VA, Veronesi R (1976). Leishmaniose tegumentar Americana. Doenças infecciosas e parasitárias.

